# Clinical Characteristics of Adult Fevered COVID-19 Patients and Predictors for Developing Severe Events

**DOI:** 10.3389/fmed.2020.00324

**Published:** 2020-07-03

**Authors:** Guyi Wang, Quan Zhang, Chenfang Wu, Fang Wu, Bo Yu, Jianlei Lv, Siye Zhang, Guobao Wu, Yanjun Zhong

**Affiliations:** ^1^Critical Care Medicine, The Second Xiangya Hospital, Central South University, Changsha, China; ^2^Critical Care Medicine, The First Hospital of Changsha, Changsha, China; ^3^Department of Oncology, The Second Xiangya Hospital, Central South University, Changsha, China

**Keywords:** COVID-19, adult, fever, C-reactive protein, lymphocytes

## Abstract

**Aim:** Clinical findings indicated that a fraction of coronavirus disease 2019 (COVID-19) patients did not show fever. However, the difference between the clinical characteristics of fevered and non-fevered patients is still unclear. The aim of the present study was to describe the clinical characteristics of these patients and analyze the predictors for severe events of adult fevered COVID-19 patients.

**Methods:** Clinical and laboratory data of fevered and non-fevered COVID-19 patients in Changsha, China, were collected and analyzed. Logistic regression analysis and Receiver Operating Characteristic Curve (ROC Curve) analysis were adopted to analyze risk factors and evaluate the effectiveness of the predictors for severe events in adult fevered COVID-19 patients.

**Results:** Of the 230 adult COVD-19 patients in this study, 175 patients (76.1%) had fever and 55 patients (23.9%) did not have fever. Compared with non-fevered patients, the fevered patients showed a lower lymphocyte proportion (*P* = 0.000) and lymphocyte count (*P* = 0.000) as well as higher levels of C-reactive protein (CRP) (*P* = 0.000) and erythrocyte sedimentation rate (*P* = 0.000). The proportion of severe cases was significantly elevated in adult fevered patients (*P* = 0.000). Compared to non-severe fevered patients, severe fevered patients showed a lower lymphocyte count (*P* = 0.000), a lower lymphocyte proportion (*P* = 0.000), and higher levels of CRP (*P* = 0.000). As determined by the multivariate analysis, CRP (OR 1.026, *P* = 0.018) and lymphocyte proportion (OR 0.924, *P* = 0.009) were significantly associated with the risk of developing severe events in fevered adult COVID-19 patients. Furthermore, ROC Curve analysis revealed that the area under the curve (AUC) for CRP combined with lymphocyte proportion to diagnose severe events in fevered adult COVID-19 patients was 0.874 (95% CI 0.820–0.927).

**Conclusions:** Adult fevered COVID-19 patients were more likely to progress into severe cases, while CRP and lymphocyte proportion were effective predictors for developing severe events in these patients.

## Introduction

The emergence of coronavirus disease 2019 (COVID-19), which was caused by severe acute respiratory syndrome coronavirus 2 (SARS-CoV-2), was first reported in China in late 2019 (1–5) and has quickly led to outbreaks in other countries, such as Italy, Iran, and South Korea (6–8). As of April 12, 2020, more than 1.6 million people worldwide have been diagnosed with COVID-19, and about 100,000 people have died (9). How to block transmission of SARS-CoV-2, early screening of severe cases, and find effective treatments are urgent issues for scientists worldwide.

As the most common symptom and sign of infection, fever is generally initiated by a pyrogen, which causes a rise in temperature setting point and increases body heat production (10, 11). Body temperature screening is widely used as a screening tool for patients with COVID-19 in many places, such as communities and airports (12). The latest report found that fever was the most common symptom of COVID-19 patients, but more than 10% of patients with COVID-19 did not develop fever (4, 13). However, differences in clinical characteristics and prognosis between fevered and non-fevered COVID-19 patients remain unclear. In this study, we presented the clinical characteristics of these patients and analyzed predictors for developing severe events through Logistic regression analysis.

## Materials and Methods

### Participants

Inclusion criteria: all laboratory-confirmed adult COVID-19 patients admitted to Public Health Treatment Center of Changsha, China, on admission from January 17 to March 15, 2020, were enrolled.

Two of our team carefully collected and reviewed the medical records of patients, individually. The detailed information on demographic data, underlying comorbidities, symptoms before and during admission, first laboratory and chest computed tomographic (CT) scans results after admission were recorded.

The temperature and symptoms before admission were based on the patient's description. After admission, all patients were monitored for axillary temperature more than once a day using mercury thermometers for 10 min each time, which were evaluated by professional nursing staff finally. Fever was defined as axillary temperature ≥37.3°C (14, 15).

### Definition and Study Endpoints

We used one of the following criterial to determine the severe cases of COVID-19: (1) respiratory rate ≥ 30 /min; (2) oxygen saturation <93%; (3) PaO_2_/FiO_2_ ≤ 300 mmHg; (4) lung lesion progression > 50% within 24–48 h; (5) mechanical ventilation was implemented; (6) shock; and (7) intensive care unit admission (16). SARS-CoV-2 nucleic acid tests were performed at least two times consecutively after remission of symptom (sampling time interval is at least 1 day). Two consecutive negative results are considered negative for the virus (16); virus shedding duration was defined as the time between symptom onset (the day of diagnosis for asymptomatic patients) and the first negative samples without any positive sample thereafter. Respiratory symptoms were defined as cough, expectoration, hemoptysis, and dyspnea, while digestive symptoms were defined as anorexia, nausea, vomiting, abdominal pain, and diarrhea.

### Statistical Analysis

We used median with range and Mann-Whitney test to depict and analyze all continuous variables because of non-normal distribution. The χ^2^ test and or Fisher's exact test was utilized to compare the differences of the categorical variables. Logistic regression analysis and Receiver Operating Characteristic Curve (ROC curve) analysis were adopted to analyze predictors and evaluate the effectiveness of the predictors for severe events in fevered COVID-19 patients. All analyses were performed using IBM SPSS version 26 software.

## Results

### Characteristics of Fevered Patients

All 230 adult patients diagnosed as COVID-19 by March 15, 2020, were included in this study; of these, 175 patients (76.1%) had fever and 55 patients (23.9%) did not have fever.

The clinical characteristics of the non-fevered and fevered patients were summarized in Table 1. There was no significant difference in age (*P* = 0.353) and gender (*P* = 0.440) between fevered and non-fevered COVID-19 patients as well as common underlying diseases, such as hypertension (*P* = 0.796), diabetes (*P* = 1.000), and cardiovascular disease (*P* = 1.000). Compared with non-fevered patients, the fevered patients showed higher ratios of respiratory symptoms (88.6 vs. 65.5%, *P* = 0.000; Table 1).

**Table 1:** Baseline characteristics of fevered and non-fevered COVID-19 patients.

	**No fever**	**Fever**	***P*-value**
	**(*n* = 55)**	**(*n* = 175)**	
Sex (male/female)	25/30	90/85	0.440
Age, median (range), y	41 (19–81)	46 (21–84)	0.353
Tobacco (*n*, %)	7 (12.7)	12 (6.9)	0.272
Alcohol (*n*, %)	3 (5.5)	7 (4.0)	0.934
Comorbidities			
Hypertension (*n*, %)	8 (14.5)	28 (16.0)	0.796
Cardiovascular disease (*n*, %)	2 (3.6)	7 (4.0)	1.000
Diabetes (*n*, %)	4 (7.3)	11 (6.3)	1.000
COPD (*n*, %)	0 (0.0)	1 (0.6)	1.000
Cerebrovascular disease (*n*, %)	0 (0.0)	6 (3.4)	0.340
Chronic liver disease (*n*, %)	2 (3.6)	10 (5.7)	0.797
Malignancy (*n*, %)	2 (3.6)	0 (0.0)	0.056
Symptoms			
Respiratory symptoms (*n*, %)	36 (65.5)	155 (88.6)	**0.000**
Digestive symptoms (*n*, %)	31 (56.4)	119 (68.0)	0.114
Chest CT positive rate (*n*, %)	50 (90.9)	170 (97.1)	0.110
Chest CT with ground-glass change	23 (41.8)	86 (49.1)	0.343
Respiratory support			
Non-invasive ventilation (*n*, %)	0 (0.0)	4 (2.3)	0.057
High-flow oxygen therapy (*n*, %)	0 (0.0)	13 (7.4)	0.081
Invasive ventilation (*n*, %)	0 (0.0)	4 (2.3)	0.575
ECMO (*n*, %)	0 (0.0)	3 (1.7)	1.000
Prognostic indicators			
Severe cases (*n*, %)	0 (0.0)	45 (25.7)	**0.000**
Length of hospital stay, median (range), days	15 (5–34)	16 (5–41)	0.424
Virus shedding duration, median (range), days	18 (3–46)	18 (6–59)	0.563
Mortality (*n*, %)	0 (0.0)	2 (1.1)	1.000

*P < 0.05 was considered statistically significant (marked in bold). Continuous variables were described as median with range and analyzed by Mann-Whitney test. Categorical variables were described as percentages and analyzed by the χ^2^ test or Fisher's exact test*.

*Abbreviations: COVID-19, coronavirus disease 2019; COPD, chronic obstructive pulmonary disease; ECMO, extracorporeal membrane oxygenation*.

The fevered COVID-19 patients had a lower lymphocyte proportion (median, 25.0 vs. 31.7%, *P* = 0.000), lower lymphocyte counts (median, 1.0 vs. 1.5 × 10^9^/L, *P* = 0.000), higher levels of C-reactive protein (CRP) (median, 20.2 vs. 3.8 mg/L, *P* = 0.000), higher erythrocyte sedimentation rate (ESR) levels (median, 48.0 vs. 27.0 U/L, *P* = 0.000), and a higher proportion of elevated procalcitonin (30.9 vs. 16.4%, *P* = 0.036; Table 2).

**Table 2:** Laboratory findings of fevered and non-fevered COVID-19 patients.

	**Normal range**	**No fever**	**Fever**	***P*-value**
		**(*n* = 55)**	**(*n* = 175)**	
White blood cell count, × 10^9^/L, median (range)	4-10	5.0 (2.4-9.9)	4.5 (0.8-13.4)	0.136
Lymphocyte count, × 10^9^/L, median (range)	0.8-4.0	1.5 (0.4-3.7)	1.0 (0.1-3.2)	**0.000**
Lymphocyte %, median (range)	20-40	31.7 (8.6-61.1)	25.0 (2.1-54.1)	**0.000**
Alanine aminotransferase, U/L, median (range)	0-42	16.9 (2.6-87.7)	20.3 (4.9-93.7)	0.058
Aspartate aminotransferase, U/L, median (range)	0-37	22.3 (11.5-78.8)	24.8 (2.0-82.1)	**0.008**
Total bilirubin, μmol/L, median (range)	3.4-20.5	10.6 (5.7-38.2)	11.0 (4.0-162.1)	0.767
C reactive protein, mg/L, median (range)	0-8	3.8 (0.2-62.1)	20.2 (0.1-101.9)	**0.000**
Erythrocyte sedimentation rate, mm/h, median (range)	0-15	27.0 (1.0-121.0)	48.0 (5.0-143.0)	**0.000**
Procalcitonin, ≥0.05 ng/ml, No. (%)	<0.05	9 (16.4)	54 (30.9)	**0.036**
Creatinine, μmol/L, median (range)	21.5-104	53.3 (25.5-124.1)	51.6 (20.6-255.7)	0.242
Creatine kinase, U/L, median (range)	10-190	68.2 (23.0-235.0)	76.5 (11.3-986.4)	0.066
Creatine kinase-MB, U/L, median (range)	0-24	9.5 (0.7-19.9)	9.6 (0.3-221.7)	0.976

*P < 0.05 was considered statistically significant (marked in bold). Continuous variables were described as median with range and analyzed by Mann-Whitney test. Categorical variables were described as percentages and analyzed by the χ^2^ test or Fisher's exact test*.

*Abbreviations: COVID-19, coronavirus disease 2019*.

Although all patients on ventilator had fever, there was no significant difference in the proportion of receiving different respiratory support between the fevered and non-fevered COVID-19 patients. In terms of prognosis indicators, there were no obvious differences in mortality (*P* = 1.000), length of hospital stay (*P* = 0.424) and virus shedding duration (*P* = 0.563) between fevered and non-fevered patients. However, fevered patients showed significantly increased proportion of severe cases (25.7 vs. 0.0%, *P* = 0.000) compared to non-fevered COVID-19 patients (Table 1).

### Characteristics of Severe Fevered Patients

Because of the significant increased probability of severe events in fevered COVID-19 patients, we further performed a subgroup analysis of fevered patients based on the disease severity. Compared to non-severe fevered patients, severe fevered patients were older (median, 57 vs. 42 years, *P* = 0.000) and had a higher proportion of underlying diseases, including hypertension (33.3 vs. 10.0%, *P* = 0.000) and cardiovascular disease (11.1 vs. 1.5%, *P* = 0.017). More severe fevered patients had respiratory symptoms (100.0 vs. 84.6%, *P* = 0.005) and ground-glass change in chest CT (68.9 vs. 42.3%, *P* = 0.002; Table 3). Severe patients also showed a lower lymphocyte count (median, 0.7 vs. 1.1 × 10^9^/L, *P* = 0.000), lower lymphocyte proportion (median, 18.6 vs. 27.5 × 10^9^/L, *P* = 0.000), and higher levels of CRP (median, 40.8 vs. 16.0 mg/L, *P* = 0.000; Table 4).

**Table 3:** Baseline characteristics of fevered COVID-19 patients with different severity.

	**Non-severe**	**Severe**	***P*-value**
	**(*n* = 130)**	**(*n* = 45)**	
Sex (male/female)	64/66	26/19	0.323
Age, median (range), y	42 (21–84)	57 (25–75)	**0.000**
Tobacco (*n*, %)	10 (7.7)	2 (4.4)	0.689
Alcohol (*n*, %)	4 (3.1)	3 (6.7)	0.537
Comorbidities			
Hypertension (*n*, %)	13 (10.0)	15 (33.3)	**0.000**
Cardiovascular disease (*n*, %)	2 (1.5)	5 (11.1)	**0.017**
Diabetes (*n*, %)	7 (5.4)	4 (8.9)	0.632
COPD, (*n*, %)	0 (0.0)	1 (2.2)	0.257
Cerebrovascular disease (*n*, %)	4 (3.1)	2 (4.4)	0.648
Chronic liver disease (*n*, %)	7 (5.4)	3 (6.7)	1.000
Malignancy (*n*, %)	0 (0.0)	0 (0.0)	NA
Symptoms			
Respiratory symptom (*n*, %)	110 (84.6)	45 (100.0)	**0.005**
Digestive symptom (*n*, %)	86 (66.2)	33 (73.3)	0.374
Chest CT positive rate (*n*, %)	125 (96.2)	45 (100.0)	0.330
Chest CT with ground-glass change	55 (42.3)	31 (68.9)	**0.002**
Respiratory support			
Non-invasive ventilation (*n*, %)	0 (0.0)	4 (8.9)	**0.004**
High-flow oxygen therapy (*n*, %)	0 (0.0)	13 (28.9)	**0.000**
Invasive ventilation (*n*, %)	0 (0.0)	4 (8.9)	**0.004**
ECMO (*n*, %)	0 (0.0)	3 (6.7)	**0.016**
Prognostic indicators			
Length of hospital stay, median (range), days	15.5 (5.0-41.0)	21.0 (8.0–41.0)	**0.007**
Virus shedding duration, median (range), days	16.5 (6.0-53.0)	21.0 (12.0–59.0)	**0.002**
Mortality (*n*, %)	0 (0.0)	2 (4.4)	0.065

*P < 0.05 was considered statistically significant (marked in bold). Continuous variables were described as median with range and analyzed by Mann-Whitney test. Categorical variables were described as percentages and analyzed by the χ^2^ test or Fisher's exact test*.

*Abbreviations: COVID-19, coronavirus disease 2019; COPD, chronic obstructive pulmonary disease; ECMO, extracorporeal membrane oxygenation. NA, not applicable*.

**Table 4:** Laboratory findings of fevered COVID-19 patients with different severity.

	**Normal range**	**Non-severe**	**Severe**	***P*-value**
		**(*n* = 130)**	**(*n* = 45)**	
White blood cell count, × 10^9^/L, median (range)	4-10	4.5 (1.9-13.4)	4.6 (0.8-10.4)	0.604
Lymphocyte count, × 10^9^/L, median (range)	0.8-4.0	1.1 (0.4-3.2)	0.7 (0.1-1.9)	**0.000**
Lymphocyte %, median (range)	20-40	27.5 (6.8-54.1)	18.6 (2.1-34.2)	**0.000**
Alanine aminotransferase, U/L, median (range)	0-42	19.7 (4.9-58.4)	23.9 (8.2-93.7)	**0.010**
Aspartate aminotransferase, U/L, median (range)	0-37	23.3 (2.0-59.5)	30.6 (16.9-82.1)	**0.000**
Total bilirubin, μmol/L, median (range)	3.4-20.5	10.9 (4.0-31.6)	11.8 (4.7-162.1)	0.805
C reactive protein, mg/L, median (range)	0-8	16.0 (0.1-91.4)	40.8 (2.7-101.9)	**0.000**
Erythrocyte sedimentation rate, mm/h, median (range)	0-15	46.0 (5.0-143.0)	51.5 (9.0-129.0)	0.208
Procalcitonin, ≥0.05 ng/ml, No. (%)	<0.05	38 (29.2)	16 (35.6)	0.429
Creatinine, μmol/L, median (range)	21.5-104	51.3 (20.6-105.1)	53.4 (21.9-255.7)	0.720
Creatine kinase, U/L, median (range)	10-190	69.2 (11.3-365.3)	92.0 (17.4-986.4)	**0.014**
Creatine kinase-MB, U/L, median (range)	0-24	9.3 (0.3-221.7)	11.2 (1.1-35.2)	**0.049**

*P < 0.05 was considered statistically significant (marked in bold). Continuous variables were described as median with range and analyzed by Mann-Whitney test. Categorical variables were described as percentages and analyzed by the χ^2^ test or Fisher's exact test*.

*Abbreviations: COVID-19, coronavirus disease 2019*.

Moreover, significantly more severe fevered patients received mechanical ventilation (non-invasive:8.9 vs. 0%, *P* = 0.004; high-flow oxygen therapy: 28.9 vs. 0%, *P* = 0.000; invasive: 8.9 vs. 0%, *P* = 0.001) and extracorporeal membrane oxygenation (ECMO) (6.7 vs. 0%, *P* = 0.016) as compared with non-severe cases. Length of hospital stay and virus shedding duration were both prolonged in severe fevered patients (Table 3).

### Analysis of Relative Factors for Severe Events

The associations between severe events of adult fevered COVID-19 patients and related factors were presented in Table 5. As determined by the multivariate analysis, CRP (OR 1.026, 95% CI 1.004–1.048, *P* = 0.018) and lymphocyte proportion (OR 0.924, 95% CI 0.871–0.980, *P* = 0.009) were significantly associated with the risk of developing severe events in fevered adult COVID-19 patients (Table 5).

**Table 5:** Logistic regression analysis for severe events of fevered COVID-19 patients.

**Variables**	**Univariate**		**Multivariate**	
	**Odds ratio (95% CI)**	***P*-value**	**Odds ratio (95% CI)**	***P*-value**
Age	1.046 (1.021-1.073)	**0.000**	1.002 (0.965-1.040)	0.921
Gender	1.411 (0.712-2.797)	0.324	1.082 (0.421-2.780)	0.870
Hypertension	4.500 (1.935-10.466)	**0.000**	2.420 (0.761-7.700)	0.135
Cardiovascular disease	8.000 (1.494-42.831)	**0.015**	2.899 (0.267-31.439)	0.381
Lymphocyte %	0.878 (0.834-0.924)	**0.000**	0.924 (0.871-0.980)	**0.009**
Creactive protein	1.042 (1.025-1.058)	**0.000**	1.026 (1.004-1.048)	**0.018**
Alanine aminotransferase	1.045 (1.015-1.076)	**0.003**	1.000 (0.953-1.050)	0.990
Aspartate aminotransferase	1.079 (1.043-1.117)	**0.000**	1.018 (0.966-1.074)	0.498
Creatine kinase	1.006 (1.003-1.010)	**0.000**	1.004 (1.000-1.008)	0.053

*P < 0.05 was considered statistically significant (marked in bold). COVID-19, coronavirus disease 2019*.

### Predictive Factor Analysis

**Figure 1:**
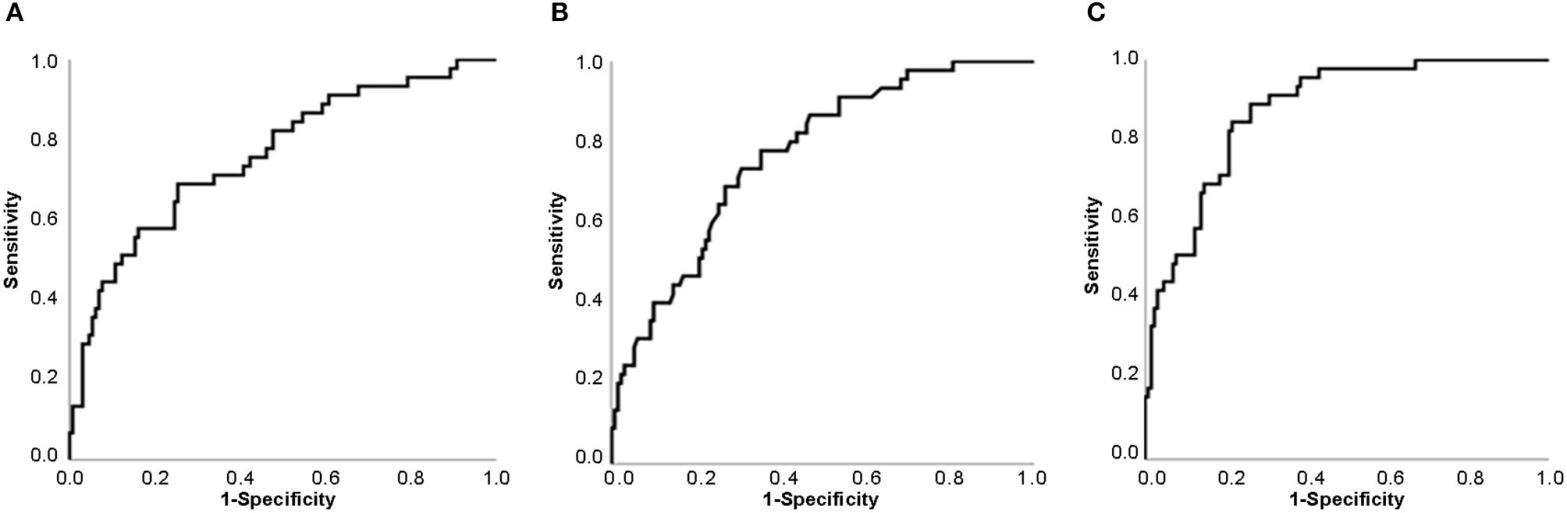
Receiver Operating Characteristic Curve (ROC Curve) for predicting the possibility of severe events in adult fevered COVID-19 patients. **(A)** ROC Curve of CRP. **(B)** ROC Curve of lymphocyte proportion. **(C)** ROC Curve of CRP combined with lymphocyte proportion. Abbreviations: CRP, C-reactive protein; COVID-19, coronavirus disease 2019.

ROC Curve analysis revealed that the areas under the curve (AUCs) for CRP and lymphocyte proportion to diagnose severe events in fevered adult COVID 19 patients were 0.759 (95% CI 0.675–0.843) and 0.767 (95% CI 0.691–0.843), respectively, while the AUC increased to 0.874 (95% CI 0.820–0.927) when CRP combined with lymphocyte proportion (Figure 1).

## Discussion

In this study, we first presented the clinical characteristics of fevered and non-fevered patients, and found that adult fevered COVID-19 patients were more likely to develop into severe cases, while CRP and lymphocyte proportion were effective predictors for developing severe events in these patients.

Although fever is the most common symptom of COVID-19 patients (5, 17–19), the proportion of fever is significantly lower compared to Middle East respiratory syndrome (MERS) and the severe acute respiratory syndrome (SARS) (20, 21). The proportion of fever varied in different studies, but a latest meta-analysis of 19 articles reported that about 88.7% COVID-19 patients had fever, which indicated that more than 10% patients had a normal temperature. In this study, we presented a slightly lower proportion of fever (76.1%) in COVID-19 patients, which may be related to the inclusion of some asymptomatic infections in this study. In general, a small number of COVID-19 patients do not show symptoms of fever, which suggests that using body temperature to screen COVID-19 patients may lead to some missed diagnosis. Nucleic acid testing now used in many places may be a more effective way to screen SAR-CoV-2 infection than body temperature testing.

Previous studies found that the elderly patients and old animal models showed an impaired fever response to infection or inflammatory factors (22–25). However, in this study, we found there was no significant difference in gender, age, and basic diseases between COVID-19 patients with fever and without fever, which suggested that the basic status may not be a major factor for fever response in COVID-19 patients.

Several studies showed the incidence of fever was significant higher in severe COVID-19 patients (26–28). In this study, we found that the proportion of severe cases was significantly higher in fevered COVID-19 patients compared to non-fevered patients, which indicated that fevered patients were more likely to progress into severe cases. Moreover, it seemed that all severe cases appeared in fevered COVID-19 patients in this study, while those non-fevered patients rarely developed into severe cases, and Huang et al. also showed none of non-fevered COVID-19 patients in their study progressed into severe cases (28), which suggested normal body temperature may be a valuable indicator to rule out severe COVID-19 patients.

Currently, hyperinflammation is considered to be an important cause of organ damage and even death in COVID-19 patients (29–32). Patients with severe or fatal COVID-19 showed significantly higher levels of inflammatory factors, such as CRP, interleukin-6 (IL-6), interleukin-2 (IL-2) and interleukin-7 (IL-7) (3, 33). In this study, fevered COVID-19 patients had a stronger inflammatory response than non-fevered patients, manifested by significantly increased erythrocyte sedimentation rate and CRP, which may be the reason for the higher proportion of severe patients in fevered COVID-19 patients.

Although the proportion of severe cases of fevered COVID-19 patients in this study increased significantly, some fevered patients did not develop severe cases. Therefore, we compared the clinical characteristics of non-severe and severe fevered patients to try to find the risk factors for severe events of fevered patients. Through logistic regression analysis, CRP and lymphocyte proportion were considered to be effective predictive factors for severe cases.

This study presented several limitations. First, few cases were included in this study, and the conclusion of the study may need to be verified by studies with larger sample size. Second, the body temperature was not measured regularly before admission, which may result in some fevers not being detected. Third, we have not obtained screening data on bacterial infection and drug treatment data after admission. Therefore, it is still unclear whether the fever after admission was caused by secondary infection, drug fever, or factors other than SAR-CoV-2 infection, and this still needs to be further studied and followed up on. Additionally, although the two died patients were both with fever, there was no statistical difference in mortality between patients with fever and those without fever, which may be relate to the low overall mortality rate and small sample size.

## Conclusions

In summary, we presented for the first time the clinical features of COVID-19 patients with or without fever. In this study, a few adult COVID-19 patients showed no fever. Adult fevered COVID-19 patients presented a higher risk of developing severe events compared to non-fevered patients, while CRP and lymphocyte proportion may be effective predictive factors.

## Data Availability Statement

The datasets presented in this article are not readily available because the data used in this paper are from public health treatment center, which can only be obtained with the approval of relevant institutions. Requests to access the datasets should be directed to Yanjun Zhong, zhongyanjun@csu.edu.cn.

## Ethics Statement

The studies involving human participants were reviewed and approved by the institutional ethics board of the Second Xiangya Hospital of Central South University (No. 2020001). Written informed consent for participation was not required for this study in accordance with the national legislation and the institutional requirements.

## Author Contributions

GWa and QZ were involved in study design, interpreting data, statistical analysis, creating tables and figures, and writing of the manuscript. YZ was involved in interpreting data, statistical analysis, designed the research, and supervised the work. CW, FW, BY, JL, GWu, and SZ were all involved in data collection, data interpretation, and critical revisions of the manuscript. All authors contributed to the article and approved the submitted version.

## Conflict of Interest

The authors declare that the research was conducted in the absence of any commercial or financial relationships that could be construed as a potential conflict of interest.
